# Dr. Chas. T. Jackson

**Published:** 1873-10

**Authors:** 


### DR. CHAS. T. JACKSON,

The eminent chemist of Boston, has been taken to the Insane Asylum at Somerville, Mass. Dr. Jackson was one of the most important experts in the celebrated trial of Professor Webster for killing Dr. Parkman. It was he who suggested to Morton the use of sulphuric ether as an anæsthetic. —*Med. and Surg. Reporter.*

